# What set some young adults apart during the COVID-19 pandemic? Mental health trajectories, risk and protective factors in an Australian longitudinal study

**DOI:** 10.1177/00048674231223690

**Published:** 2024-01-11

**Authors:** Amarina Donohoe-Bales, Siobhan O’Dean, Scarlett Smout, Julia Boyle, Emma Barrett, Maree Teesson, Marlee Bower

**Affiliations:** The Matilda Centre for Research in Mental Health and Substance Use, Faculty of Medicine and Health, The University of Sydney, Camperdown, NSW, Australia

**Keywords:** Young adult, mental health, trajectories, Australian population, COVID-19 pandemic

## Abstract

**Objective:**

Evidence suggests that young adults (aged 18–34) were disproportionately impacted by the COVID-19 pandemic, but little is known about their longer-term mental health changes beyond the early pandemic period. This article investigates heterogeneous trajectories of mental health among Australian young adults across 2 years of the pandemic and identifies a broad range of associated risk and protective factors.

**Method:**

Young adults (*N* = 653, M_age_ = 27.8 years) from the longitudinal Alone Together Study were surveyed biannually between July 2020 and June 2022. Measures assessed anxiety (7-item Generalised Anxiety Disorder scale) and depression (9-item Patient Health Questionnaire) symptoms at Waves 1–4, as well as demographic, psychological, adversity and COVID-19 factors at baseline.

**Results:**

Four and three distinct trajectories of anxiety and depressive symptoms, respectively, were identified through growth mixture modelling. The proportion of participants in each anxiety trajectory were Asymptomatic (45.9%), Mild Stable (17.9%), Moderate–Severe Stable (31.1%) and Initially Severe/Recovering (5.1%). For depression, Mild Stable (58.3%), Moderate–Severe Stable (30.5%) and Reactive/Recovering (11.2%). Baseline factors associated with severe symptom trajectories included a lifetime mental health disorder, pre-pandemic stressful events, identifying as LGBTQIA+ and/or female, and experiencing one or more infection-control measures. Higher household income was protective.

**Conclusion:**

Most young adults demonstrated stable trajectories of low or high symptoms during the pandemic, with smaller groups showing initially severe or reactive symptoms followed by marked improvements over time. Vulnerable subgroups (gender- or sexuality-diverse, those with prior adversity or pre-existing mental ill-health) may face ongoing impacts and require targeted psychosocial supports to assist their mental health recovery post-COVID-19 and in the event of future crises.

## Introduction

There is growing evidence that the COVID-19 pandemic disproportionately impacted the mental health of young people, particularly adolescents and emerging adults, compared to older age groups (Australian Institute of Health Welfare, 2021; Bower et al., 2023; Santomauro et al., 2021). The large-scale psychological effects and socioeconomic disruptions associated with the pandemic are expected to leave a lasting toll on young peoples’ mental health (Coker et al., 2023). While long-term adolescent (aged 13–18) mental health outcomes have been closely tracked during the pandemic (Thorisdottir et al., 2023), there has been limited evaluation of young adults’ (aged 18–34) mental health, including patterns of change throughout the pandemic and related risk and protective factors (Creswell, 2023).

Young adulthood (defined here as 18–34 years) is a critical period of development characterised by rapid personal development and major life transitions, such as entering the workforce, living independently, achieving greater autonomy and assuming more adult roles and responsibilities (Wood et al., 2018). Young adults may be particularly vulnerable to pandemic-related challenges, including social disruptions and financial insecurity related to infection-control measures. Furthermore, early adulthood is a period of increased vulnerability for the emergence of psychopathologies and poor psychosocial adjustment, with approximately two-thirds of diagnosed mental disorders occurring before the age of 25 (Australian Bureau of Statistics, 2022; McGrath et al., 2023; Solmi et al., 2022). Previous pandemics and major economic crises have shown that associated mental health effects persist well beyond the acute crisis period (Newnham et al., 2022). Yet, much existing evidence on the mental health impacts of the pandemic on young adults focuses on the first 6–12 months of the crisis (Villanti et al., 2022). Examining longer-term mental health among young adults is therefore crucial for understanding and mitigating the impacts of the pandemic and future health emergencies (Wiedemann et al., 2022).

Elevated levels of depression, anxiety, psychological distress, suicidal ideation and loneliness among young adults during the pandemic are well documented (Batterham et al., 2022; Horigian et al., 2021; Varma et al., 2021). However, a vast majority of studies have adopted cross-sectional designs conducted shortly after the initial onset of the pandemic. The few existing longitudinal studies predominantly occurred during the early stages of the pandemic between 2020 and early 2021, with limited timepoints. Nonetheless, these studies do show that mental health markedly deteriorated among young adults compared to pre-pandemic levels (Botha et al., 2022a; Daly et al., 2022; Edwards et al., 2020; Upton et al., 2023). Moreover, these study designs assume that young adults’ mental health followed a single, linear worsening trajectory and have limited exploration of factors associated with varying symptom trajectories to ascertain *who* is at the highest risk of poorer outcomes.

Existing cross-sectional research among young adult populations during the pandemic suggests that certain groups may experience poorer mental health, including those that identify as female, have a pre-existing mental disorder, lower income and experience of stressful life events, but this warrants further longitudinal investigation (Haag et al., 2022; Mir et al., 2023; Shanahan et al., 2022; Stroud and Gutman, 2021; Wiedemann et al., 2022). In addition, the pandemic and associated containment measures exacerbated other key social determinants of mental health and structural drivers of health inequities more broadly, including economic stability, employment, housing, social isolation and public policies (Bernardini et al., 2021). For example, pandemic-induced job insecurity, economic hardship, social disconnection and loneliness markedly worsened emotional distress, well-being and substance use among young adult populations (Graupensperger et al., 2023; Shanahan et al., 2022; Varma et al., 2021). Broader infection-control measures (e.g. social distancing and lockdowns) and COVID-19 infection were also associated with mental ill-health in younger populations (Graupensperger et al., 2023; Thorpe and Gutman, 2022). However, it remains unclear whether pre-pandemic and mid-pandemic social determinants of mental health – referring to demographic characteristics and COVID-19-related stressors, respectively – are associated with both short- *and* longer-term psychological distress throughout the pandemic period, beyond 2020. It is well documented that even short-lived changes to social contexts during youth can have long-term effects on mental health into adulthood and across the life course (Braveman and Gottlieb, 2014; Viner et al., 2012), with recent evidence suggesting that early-stage social and economic disruptions during the pandemic may place young people at risk for longer-term distress and mental health disorders (Australian Institute of Health Welfare, 2021; Curtis et al., 2023). Indeed, the combination of acute and unprecedented disruptions to employment, income, housing and social connection, as well as the high level of daily threat, during the early stages of the pandemic created conditions under which maladjustment and persistent poor mental health were likely to develop. Examining the association between pre-pandemic and mid-pandemic social determinants and longer-term mental health trajectories will provide critical information to design effective interventions and policies to mitigate mental health disparities among young adults.

This study aims to identify distinct trajectories of anxiety and depression symptoms among Australian young adults (aged 18–34) during the pandemic between July 2020 and June 2022, and establish whether social determinants – including sociodemographic characteristics, psychological and adversity factors, and COVID-19-related stressors – measured at baseline predict trajectory membership. Our exploratory study focuses on Australian young adults aged 18–34 years, informed by age ranges used to represent young adulthood in prior COVID-19 literature (Botha et al., 2022a; Upton et al., 2023), as well as young adult age ranges used by the Australian Bureau of Statistics (2013). Exploring longer-term mental health trajectories during the pandemic, as well as risk and protective factors predicting trajectory membership, will illuminate prevention, targeted early intervention and policy strategies and levers to promote young adult psychosocial well-being during future crises.

## Methods

### Participants

Data were drawn from the longitudinal *Alone Together Study*: a five-wave, 30-month, Australia-wide research project investigating the mental health impacts of the COVID-19 pandemic and associated personal, social and economic changes among a community sample of Australian adults (18 years and older). Ethics approval was obtained from the University of Sydney’s Human Research Ethics Committee (HREC; 2020/460). This study analysed data from a subsample of young adults (*N* = 653) who completed baseline. Four surveys were administered biannually between 2020 and 2022: baseline data collection commenced during July to December 2020 (Wave 1); follow-up survey 1 during January to June 2021 (Wave 2); follow-up survey 2 during July to December 2021 (Wave 3); and follow-up survey 3 during January to June 2022 (Wave 4). Recruitment, eligibility, consent and participant reimbursement protocols are detailed in Appendix S2.

A Youth Advisory Board (YAB), comprised of a diverse group of 10 young people ages 16–25 years, from the Matilda Centre for Research in Mental Health and Substance Use was consulted to gain feedback on this study’s aims and methodological design (Prior et al., 2022). Input from the YAB facilitated further development and refinement of research questions and variables of interest, thus strengthening the overall quality, validity and relevance of this study in relation to the lived experience of Australian young adults. Ethical approval was not required, as YAB members were engaged in an expert and advisory capacity, not as study participants.

### Measures

#### Mental health outcomes

The 7-item Generalised Anxiety Disorder scale (GAD-7) and 9-item Patient Health Questionnaire (PHQ-9) are self-report measures assessing the frequency and severity of anxiety and depression symptoms, respectively, in the previous 2 weeks (Kroenke et al., 2001; Spitzer et al., 2006). Higher scores on the GAD-7 (range = 0–21) and PHQ-9 (range = 0–27) indicate greater functional impairment, disability and symptom-related difficulty. The GAD-7 and PHQ-9 demonstrate excellent internal consistency at each timepoint (>α = 0.92 and >α = 0.90, respectively) and strong validity, and are closely aligned with diagnostic criteria for generalised anxiety disorder and major depressive disorder (Kroenke et al., 2010). Clinical cut points of 5, 10 and 15 represent mild, moderate and severe symptom levels for both scales (Kroenke et al., 2010). GAD-7 and PHQ-9 were assessed at Waves 1–4 to inform anxiety and depression trajectories over time.

#### Predictors of trajectories

Data on risk and protective factors for depression and anxiety symptoms were collected at Wave 1/baseline only. This study applied a social determinants of mental health framework to guide the selection of potential risk and protective factors to recognise the complex interplay between individual, social and societal forces at play during the pandemic context (Jayasinghe, 2015; World Health Organization, 2023). Social determinants included pre-pandemic individual-level demographic characteristics, psychological and adversity factors, as well as mid-pandemic stressors, such as changes to income, housing, employment, social support and government policies.

Sociodemographic factors included age; self-reported gender identity (female, male, non-binary, other); gender and sexual diversity (lesbian, gay, bisexual, transgender, queer, intersex, asexual (LGBTQIA+) versus not LGBTQIA+); educational level completed (no tertiary education, trade or technical, university); current household income (responses ranging from <$300 per week to >$2700 per week); and state/territory of residence.

Psychological and adversity factors included a lifetime diagnosis of a mental health disorder (binary coded as yes/no) and exposure to stressful events in the 12 months prior to COVID-19 (henceforth ‘pre-pandemic stressful events’), using the List of Threatening Experiences Questionnaire (LTE-Q) (Brugha and Cragg, 1990). The LTE-Q measures stressful events (total score range = 0–15), such as illness, financial crisis and relational problems, with high test–retest reliability (kappa range = 0.61–0.87) (Motrico et al., 2013).

COVID-19-related stressors are detailed in Table 1. Measured at Wave 1/baseline only, these spanned a broad range of stressors that occurred since the pandemic began, including disruptions to income, employment, housing and social belonging, as well as experiences of infection-control measures and national-level stringency and containment policies.

**Table 1.** COVID-19-related stressors measured at baseline only.

	Descriptions
Income disruptions since COVID-19	Calculated as the difference between pre- and mid-pandemic household income. Pre- and mid-pandemic response categories included *<*$300 per week; $300–$575 per week; $575–$1075 per week; $1075–$1700 per week; $1700–$2400 per week; *>*$2400 per week. Change scores were categorised as a ‘reduction in income’, ‘increase in income’ or ‘no change’.
Employment disruptions since COVID-19	Binary coded as ‘no disruptions’ or ‘one or more disruptions’ based on indicators of pandemic-related work disruptions, including job loss, loss of hours or being forced or asked to take paid or unpaid leave.
Housing disruptions since COVID-19	Binary coded as ‘no disruptions’ or ‘one or more disruptions’ based on indicators of pandemic-related housing disruptions, including a change in household structure, moving or relocating or becoming homeless.
Social disruptions since COVID-19	Calculated using the 4-item Exeter Identity Transitions Scales (EXITS) which examines a person’s perceived sense of belonging and connection to multiple social groups (Haslam et al., 2008). Scale items, such as ‘I belong/ed to lots of different social groups’ and ‘I have/had strong ties with lots of people’, were rated on a 5-point scale (1 = Strongly disagree, 5 = Strongly agree; total score range: 5–20). Participants provided a retrospective pre- and mid-pandemic EXITS rating and change scores were calculated. Change scores were categorised as ‘social impairment’ (i.e. a reduction in social group membership), ‘social gains’ (i.e. an increase in social group membership) or ‘no change’.
Experience of infection-control measures since COVID-19	Binary coded as ‘no experiences’ or ‘one or more experiences’ based on whether participants had a history of quarantining or self-isolating (i.e. due to returning from overseas or after having contact with a COVID-positive case) since the pandemic, either voluntarily or directed by a government health department.
National stringency index	A composite measure (between 0 and 100) based on indicators of national government responses to COVID-19 in Australia, including national closure and containment policies, economic response policies, and broader public health policies collected by the Oxford COVID-19 Government Response Tracker (OxCGRT) (Hale et al., 2020). Participant data were matched with a national stringency index score based on the corresponding date of baseline survey completion. Due to substantial missing data in the OxCGRT during the 2020 period, more detailed state-level and city-level stringency indices could not be included in the analysis and therefore do not explore state- and local stringency variations (Biddle et al., 2022).

### Statistical analysis

Missing data at follow-up timepoints were handled using full information maximum likelihood estimation in Mplus to provide unbiased estimates of data missing at random (Muthén and Muthén, 2017) (Appendix S1). Growth mixture modelling (GMM), using GAD-7 and PHQ-9 data from all four survey waves, were conducted in Mplus Version 8.9 (Muthén and Muthén, 1998-2017) to estimate distinct latent trajectories of anxiety and depression symptoms, respectively. A major advantage of GMM is its ability to identify unobserved subgroups, or ‘classes’, within a population based on similar trajectories over time that differ with respect to mean amount of change, interindividual differences in change and longitudinal patterns of change (Ram and Grimm, 2009). We tested different specifications of time scores (linear, quadradic and freely estimated) on unconditional growth models (i.e. no classes) to establish the best fitting time structure for the data. Models with increasing numbers of classes (one to six classes) were fit to the data and the optimal number of latent classes for each outcome variable were determined on the basis of parsimony, interpretability of classes and model fit statistics (Appendix S3).

Participants were customarily assigned to classes with the highest posterior probability before multinomial logistic regressions were applied to identify baseline factors associated with anxiety and depression symptom trajectories. Regressions were carried out using a stepped approach to determine which factors were uniquely associated with group membership in each trajectory. First, univariate regressions between all variables (sociodemographic, psychological, adversity, COVID-19-related) and trajectories were investigated for both outcomes. Second, all sociodemographic factors were included in a multivariate model to ascertain whether associations observed in univariate models held when controlling for other related variables. Third, psychological and adversity factors were included in a multivariate model, while controlling for sociodemographic factors. Fourth, all COVID-19-related factors were included in a multivariate model, while controlling for sociodemographic, psychological and adversity factors. Statistical significance was set at *p* < 0.05. All regression analyses were carried out using R Version 4.3.1 (R Core Team, 2022).

## Results

The analysed sample included 653 participants aged 18–34 years (M_age_ = 27.8, SD = 4.4) from the baseline survey (Wave 1, July–December 2020). Table 2 shows baseline sample characteristics. Of those 653 participants, 374 (57.3%) completed the first follow-up survey (Wave 2, January–June 2021), 399 (61.1%) completed the second (Wave 3, July–December 2022), and 322 (49.3%) completed the third (Wave 4, January–June 2022). Approximately three-quarters (73.8%) completed two or more follow-up surveys. The prevalence of clinically significant (moderate-to-severe) symptoms of anxiety and depression based on clinical cut points at baseline was 38% (*N* = 248) and 45.8% (*N* = 299), respectively (Table 3).

**Table 2.** Baseline sample characteristics of young adults in the Alone Together Study (*N* = 653).

	*N* (%)
Age	
18–24	167 (25.6)
25–34	486 (74.4)
Gender	
Female	493 (75.5)
Male	141 (21.6)
Non-binary	15 (2.3)
Other	4 (0.6)
Aboriginal and Torres Strait Islander	7 (1.1)
English as a second language	76 (11.6)
LGBTQIA+	161 (24.7)
State/Territory	
New South Wales	295 (45.2)
Victoria	218 (33.4)
Queensland	54 (8.3)
Australian Capital Territory	27 (4.1)
Western Australia	21 (3.2)
South Australia	20 (3.1)
Tasmania	16 (2.5)
Northern Territory	2 (0.3)
Current household income	
<$300 per week	24 (3.7)
$300–$575 per week	62 (9.5)
$575–$1075	98 (15.0)
$1075–$1700	139 (21.3)
$1700–$2400	121 (18.5)
>$2400	152 (23.3)
Prefer not to say	57 (8.7)
Education	
Finished year 12	609 (93.3)
No tertiary education	84 (12.9)
Completed trade/technical college	72 (11.0)
Completed university	497 (76.1)
Employed full-time, part-time or casual	401 (61.4)
Student	136 (20.8)
Unemployed	82 (12.6)
Lifetime mental health disorder	323 (49.5)
Pre-pandemic stressful events	
No events	300 (45.9)
One or more events	336 (51.5)
COVID-19-related stressors	
Income disruptions since COVID-19: Reduction in income	137 (21.0)
Income disruptions since COVID-19: Increase in income	59 (9.0)
Employment disruptions since COVID-19	196 (30.0)
Housing disruptions since COVID-19	126 (19.3)
Social disruptions since COVID-19: Social impairment	425 (65.1)
Social disruptions since COVID-19: Social gains	67 (10.3)
Experience of infection-control measures since COVID-19	214 (32.8)

LGBTQIA: lesbian, gay, bisexual, transgender, queer, intersex, asexual.

**Table 3.** Depression and anxiety symptom scores across all survey waves.

	Respondents *N* (%)	Mean score (SD)	Clinically significant symptoms^a^ *N* (%)
Anxiety (GAD-7)			
Wave 1: July–December 2020	653	8.23 (5.83)	248 (38.0)
Wave 2: January–June 2021	374	7.44 (5.85)	122 (32.6)
Wave 3: July–December 2021	399	7.98 (5.91)	144 (36.1)
Wave 4: January–June 2022	322	7.24 (5.72)	104 (32.3)
Depression (PHQ-9)			
Wave 1: July–December 2020	653	9.89 (6.67)	299 (45.8)
Wave 2: January–June 2021	374	8.74 (6.58)	144 (38.5)
Wave 3: July–December 2021	399	9.71 (6.86)	179 (44.9)
Wave 4: January–June 2022	322	8.82 (6.72)	115 (35.7)

SD: standard deviation; GAD-7: 7-item Generalised Anxiety Disorder scale; PHQ-9: 9-item Patient Health Questionnaire.

a Scores of 10+ indicate moderate to severe symptoms.

### Identification of distinct trajectories

Based on model fit statistics and class interpretability, the 4- and 3-class models were chosen for anxiety and depressive symptoms, respectively (see Appendix S3 for more details). The following analyses were exploratory in nature and were not pre-registered.

**Figure 1.**
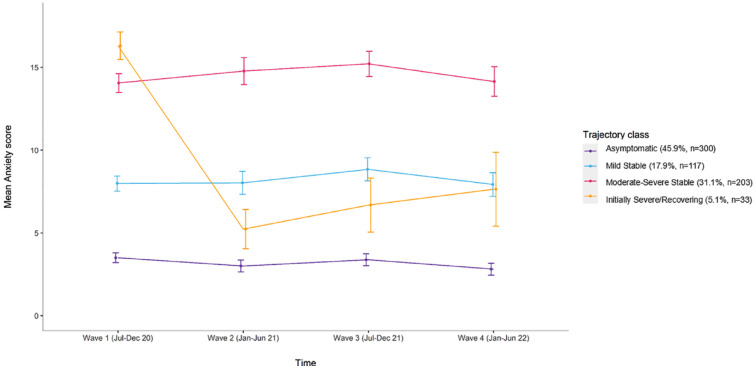
Trajectories for symptoms of anxiety (GAD-7) including raw means (with standard deviations) and *N* (%) for each class.

**Figure 2.**
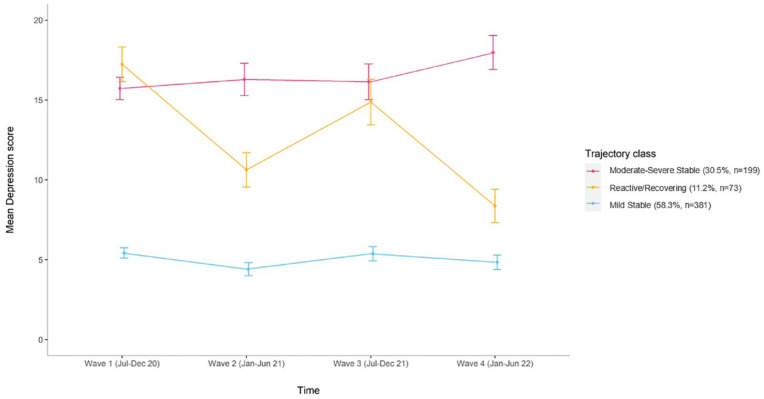
Trajectories for symptoms of depression (PHQ-9) including raw means (with standard deviations) and *N* (%) for each class.

We identified four distinct trajectories for anxiety symptoms: ‘Asymptomatic’ with consistently minimal-to-no symptoms (45.9%, *n* = 300), ‘Moderate–Severe Stable’ with consistently high symptoms (31.1%, *n* = 203), ‘Mild Stable’ with consistently low symptoms (17.9%, *n* = 117) and ‘Initially Severe/Recovering’ with initially high symptoms then declining (5.1%, *n* = 33) (Figure 1). We identified three distinct trajectories for depression symptoms: ‘Mild Stable’ with consistently low-level symptoms (58.3%, *n* = 381), ‘Moderate–Severe Stable’ with consistently high symptoms (30.5%, *n* = 199) and ‘Reactive/Recovering’ with high symptoms at Wave 1/baseline and Wave 3 during periods of high pandemic-related restrictions and lockdowns across Australia, but eventually recovered with an overall pattern of declining symptoms (11.2%, *n* = 73) (Figure 2).

### Factors associated with class membership

Participants in the ‘Asymptomatic’ and ‘Mild Stable’ anxiety and depressive symptom trajectories, respectively, were used as the reference class to examine factors associated with class membership in the more severe trajectories. For each outcome, several stepwise multivariate models were performed to determine the unique contributions of pre-pandemic participant characteristics and COVID-19-related stressors. Model 1 included all sociodemographic variables; Model 2 included all psychological and adversity factors, adjusting for sociodemographic variables; and Model 3 included all COVID-19-related stressors, adjusting for sociodemographic, psychological and adversity factors. Univariate and multivariate anxiety and depression models are in Appendix S6.

#### Anxiety

##### Model 1

The odds of being in Moderate–Severe Stable (odds ratio [OR] = 2.47; 95% confidence interval [CI] = 1.48-4.12) and Mild Stable trajectories (OR = 2.03; 95% CI = 1.11–3.71), compared to the Asymptomatic trajectory, were greater for female-identifying participants compared to male-identifying participants. In addition, the odds of being in the Moderate-Severe Stable trajectory, compared to the Asymptomatic trajectory, were greater for people who identified as LGBTQIA+ (OR = 1.74; 95% CI = 1.09–2.77).

##### Model 2

Adjusting for sociodemographic variables, having a lifetime mental health disorder was associated with greater odds of being in the Moderate–Severe Stable (OR = 3.01; 95% CI = 1.89-4.81) and Mild Stable (OR = 2.44; 95% CI = 1.45-4.09) trajectories, compared to the Asymptomatic trajectory. Furthermore, each unit increase in the number of pre-pandemic stressful events was associated with an increase in the odds (OR = 1.35; 95% CI = 1.10–1.66) of being in the Moderate–Severe Stable trajectory, compared to the Asymptomatic trajectory.

##### Model 3

After controlling for all pre-pandemic sociodemographic, psychological and adversity factors, experiencing one or more COVID-19-related infection-control measures (i.e. quarantining or self-isolating) was associated with higher odds of being in the Mild Stable trajectory (OR = 2.18; 95% CI = 1.23–3.86) compared to the Asymptomatic trajectory. In contrast, experiencing one or more infection-control measures was associated with lower odds of being in the Moderate–Severe Stable (OR = 0.57; 95% CI = 0.33–0.99); these results must be interpreted with caution due to the corresponding *p* value approaching non-significance (*p* = 0.047). Finally, those who experienced higher national-level stringency measures during the Wave 1 baseline survey had lower odds (OR = 0.81; 95% CI = 0.66–0.99) of being in the Initially Severe/Recovering trajectory compared to the Asymptomatic trajectory; again, this *p* value approached non-significance (*p* = 0.043).

#### Depression

##### Model 1

Identifying as LGBTQIA+ was associated with higher odds of being in the Moderate–Severe Stable (OR = 2.14; 95% CI = 1.38–3.32) and Reactive/Recovering (OR = 2.19; 95% CI = 1.78–4.09) trajectories, compared to the Mild Stable trajectory. Those who identified as female (OR = 2.61; 95% CI = 1.15–5.92) or non-binary (OR = 7.22; 95% CI = 1.23-42.27) had greater odds of being in the Reactive/Recovering trajectory, relative to the Mild Stable group. However, due to wide CIs and low *n* in the non-binary group, this finding must be interpreted with caution. In addition, for every increase in household income bracket, there was reduced odds of being in the Moderate–Severe Stable (OR = 0.80; 95% CI = 0.69–0.91) and Reactive/Recovering (OR = 0.81; 95% CI = 0.67–0.99) trajectories, compared to the Mild Stable trajectory. However, the *p* value approached non-significance (*p* = 0.041) in the Reactive/Recovering trajectory.

##### Model 2

Adjusting for sociodemographic variables, having a lifetime mental health disorder was associated with greater odds of being in the Moderate–Severe Stable (OR = 2.10; 95% CI = 1.35–3.27) and Reactive/Recovering (OR = 1.92; 95% CI = 1.01–3.65) trajectories, compared to the Mild Stable group. Furthermore, each unit increase in the number of pre-pandemic stressful events was associated with an increase in the odds (OR = 1.30; 95% CI = 1.07–1.58) of being in the Moderate–Severe Stable trajectory, compared to the Mild Stable trajectory.

##### Model 3

After controlling for all pre-pandemic sociodemographic, psychological and adversity factors, social gains (i.e. an increase in social group membership) since the pandemic began were associated with greater odds of being in the Moderate–Severe Stable trajectory (OR = 2.51; 95% CI = 1.00–6.30) compared to the Mild Stable trajectory. However, the corresponding *p* value (*p* = 0.49) borderlines non-significance and, again, must be interpreted with caution. Finally, young adults who experienced higher national-level stringency measures during the Wave 1 baseline survey had lower odds (OR = 0.86; 95% CI = 0.74–0.99) of being in the Reactive/Recovering trajectory compared to the Mild Stable trajectory.

## Discussion

This is the first study to examine trajectories of anxiety and depressive symptoms among Australian young adults over 24 months of the COVID-19 pandemic. The analysis identified four latent trajectories of anxiety symptoms and three trajectories of depressive symptoms, highlighting that young adults’ mental health during the pandemic was heterogeneous. Most young adults followed asymptomatic or mild stable symptom trajectories (58.3–63.9%), while a smaller group followed initially high then decreasing symptom trajectories (5.1–11.2%). These findings show that a sizable proportion of young adults in our sample were resilient and adapted to major changes and disruptions that occurred during the pandemic. Indeed, several studies have observed similar resilient trajectories among both young adult and adult populations in the first 12 months of the pandemic (Schäfer et al., 2022). Despite this, approximately one-third (30.5–31.1%) of our sample followed moderate-to-severe stable trajectories, indicative of clinically significant symptoms, which may reflect ongoing challenges that some young adults face in the late- and post-COVID-19 era. These results suggest that mental health has worsened across this group, which has indeed been supported by findings of a nationally-representative data indicating a fourfold increase in distress among young adults from 2019 to mid-pandemic in July 2020 (Botha et al., 2022a).

Notably, our findings reveal that symptom trajectories remained relatively stable, showing either consistently high or low symptoms, with limited variation over time for most young adults in our sample. We did not observe any worsening trajectories. This finding contrasts with previous studies showing mental health deterioration among young adults during the first 6 months of the pandemic (Daly et al., 2022; Upton et al., 2023). As baseline data collection for our study occurred after this acute period and captures much longer-term impacts than other Australian studies, it appears that the rapid deterioration observed in other studies may not have continued beyond the initial fear phase of the pandemic (Sun et al., 2023). This explanation is consistent with one large-scale representative study of young adult twins in the United Kingdom (Rimfeld et al., 2022), which similarly did not detect any significant overall changes in psychological distress during the pandemic, apart from an initial spike in early 2020 that recovered quickly thereafter; this may explain the initially high then decreasing anxiety and depressive symptom trajectories observed in our study. Overall, our trajectories reflect minimal changes, as well as signs of recovery, in young adults’ mental health throughout the 2-year pandemic period. However, in the absence of pre-COVID-19 data, we were unable to quantify if and how anxiety and depressive symptoms have changed from pre- to mid-pandemic in our own sample. Therefore, it is difficult to conclude if our trajectories reflect changes directly attributable to the pandemic or simply a continuation of general pre-pandemic population-level mental health patterns.

We consistently found that those who identified as LGBTQIA+ and/or as female, had a lifetime mental health disorder or experienced stressful events prior to the pandemic were at higher risk of experiencing more severe anxiety and depressive symptom trajectories. These findings are congruent with prior literature showing that young people with pre-pandemic sociodemographic vulnerabilities continued to face high levels of distress during the pandemic (Guzman Holst et al., 2023; Thorpe and Gutman, 2022). Young adults who identify as LGBTQIA+ and/or female, as well as those with a pre-existing mental health disorder and an accumulation of pre-pandemic stressful events, may require additional targeted mental health supports early during pandemics and other large-scale disasters, including Government-subsidised psychology sessions and outreach initiatives, to protect the mental health of these groups and reduce pandemic-related barriers to accessing mental health supports (Gao et al., 2023). Interestingly, we observed that individuals with a lifetime mental health disorder were more likely to belong to chronic, severe and reactive mental health trajectories, but not the Initially Severe/Recovering anxiety trajectory, when compared to the low stable symptom groups. In accordance with adult COVID-19 literature, these findings suggest that young adults with a pre-existing mental health condition may not have had the psychological resources to cope and adapt to pandemic-related stressors and changes over time (i.e. multiple lockdowns, social distancing and extended restrictions), and may have faced additional challenges such as reduced access to professional mental health support, thus hampering their mental health recovery over the course of the pandemic (Bendau et al., 2021; Neelam et al., 2021).

Only some COVID-19-related stressors were significantly associated with mental health trajectories after accounting for individual-level pre-pandemic characteristics (i.e. sociodemographic, psychological and adversity factors). Higher average national-level stringency measures at baseline (encompassing national government-initiated containment policies such as lockdowns) were associated with lower odds of being in the Initially Severe/Recovering and Reactive/Recovering mental health trajectories, compared to Asymptomatic and Mild Stable symptom groups. These findings suggest that more stringent national containment measures appeared to support mental health resilience, particularly during the early stages of the pandemic, consistent with a large systematic review (Lee et al., 2021). However, experiencing self-isolation or quarantine was associated with mildly elevated and sustained anxiety symptoms. Indeed, previous studies have demonstrated that young people who underwent COVID-19-related self-isolation experienced heightened distress associated with work and income disruptions, as well as fear of infection (Bonati et al., 2022; Kornilaki, 2022). In the event of future health emergencies, the planning of infection-control measures, such as self-isolation and quarantine, should carefully consider consequences for, and strategies to address, psychological health and safety, including sufficient access to mental health supports for vulnerable young people (Henssler et al., 2021).

Surprisingly, a COVID-19-related reduction in income was not significantly associated with worse mental health trajectories, which contradicts prior research (Argabright et al., 2022). However, we may be observing the effectiveness of the ‘Coronavirus Supplement’: a large-scale income support initiative introduced by the Federal Government in April 2020 (pre-baseline) to replace the lost income of Australians without adequate employment during the pandemic. Several studies have shown that supplemental income measures reduce both financial and mental distress particularly among economically vulnerable populations, including young adults (Botha et al., 2022b; Simpson et al., 2021). Supporting this notion, our findings also show that higher household income at baseline had a protective effect on mental health, associated with lower odds of experiencing severe depressive symptoms. Therefore, coordinated fiscal planning and supports remain an imperative social determinant of mental health and well-being as young adults continue to face cost-of-living pressures and financial stress in the fallout of COVID-19 (Australia’s Mental Health Think Tank, 2021).

All other COVID-19-related stressors were non-significant in the multivariate models, which could indicate that early COVID-19-related factors may be weakly associated with enduring mental health outcomes. Indeed, prior studies have shown that COVID-19-related stressors experienced early in the pandemic were strong predictors of immediate distress (Villanti et al., 2022). The impact of additive COVID-19-related stressors on mental health measured at multiple timepoints throughout the pandemic was not addressed in this study but remains an important priority for future research. New and evolving COVID-19-related stressors that occurred between 2020 and 2022, such as reoccurring lockdowns, shifting to working from home, fluctuating infection rates, and the winding back of the financial ‘Coronavirus Supplement’ (Botha et al., 2022b), may have contributed to the development of mental health trajectories and, as such, should be examined to contribute to the broader picture of mental health in the time of COVID-19.

### Strengths and limitations

This study possesses several strengths. The analysis utilised statistically robust GMM using four waves of data collection to assess longer-term mental health outcomes during the pandemic in an under-studied population. The current findings, spanning July 2020 to June 2022, elucidate how young adults navigated and psychologically adapted from the acute, early and unprecedented stages of the pandemic to the longer-term ‘new normal’. This study explores a broad range of risk and protective factors employing a social determinants of mental health lens to understand how early indicators in the pandemic heralded longer-term mental health outcomes, filling an important research gap identified in previous literature (Kauhanen et al., 2023).

Notwithstanding, our findings should be interpreted in the context of some further limitations. First, our sample was non-representative of the Australian young adult population. As such, the results may not be generalisable and may be prone to self-selection bias. Second, while the GAD-7 and PHQ-9 are widely validated measures of anxiety and depressive symptoms (Kroenke et al., 2010), they may be associated with self-report biases. Third, while the baseline survey commenced early in the pandemic when restrictions and knowledge around the pandemic were still emerging in Australia, the lack of pre-pandemic data limits our understanding of changes between the initial onset of the pandemic (March 2020) and Wave 1/baseline (July 2020). Fourth, LGBTQIA+ was assessed as a single category and therefore cannot account for variations within this acronym. Future research would benefit from exploring the diverse needs and experiences within this group, in light of our findings, to better support the mental health and wellbeing of LGBTQIA+ youth in the post-COVID context. Finally, the long-term impact of the pandemic on the mental health of young adults, beyond 2022, remains a critical research priority, as young adulthood represents a sensitive period for the development of psychological disorders (McGrath et al., 2023).

## Conclusion

Our findings indicate a mixed and complex picture of young adults’ mental health during the COVID-19 pandemic, with a majority showing broadly stable trajectories of low and high anxiety and depressive symptoms, with smaller but substantial trajectories showing initially severe symptoms followed by marked improvements over time. Young adults with pre-existing mental illness, recent adversity and identifying as LGBTQIA+ and/or female demonstrated the highest risk of moderate-to-severe symptoms, highlighting the importance of individual-level sociodemographic vulnerability factors. Furthermore, those who experienced one or more infection-control measures – including quarantining and self-isolation – were more likely to experience elevated anxiety. This study adds nuance to evidence surrounding mental health deterioration during the COVID-19 pandemic, showing that not all young adults experienced the pandemic equally. Findings can inform prevention and early intervention targets for both ongoing COVID-19 recovery and future-proofing ahead of other major crises, particularly among vulnerable groups (Gruber et al., 2023).

## Supplemental Material

sj-docx-1-anp-10.1177_00048674231223690 – Supplemental material for What set some young adults apart during the COVID-19 pandemic? Mental health trajectories, risk and protective factors in an Australian longitudinal study Supplemental material, sj-docx-1-anp-10.1177_00048674231223690 for What set some young adults apart during the COVID-19 pandemic? Mental health trajectories, risk and protective factors in an Australian longitudinal study by Amarina Donohoe-Bales, Siobhan O’Dean, Scarlett Smout, Julia Boyle, Emma Barrett, Maree Teesson and Marlee Bower in Australian & New Zealand Journal of Psychiatry
