# Supplementary material for: What set some young adults apart during the COVID-19 pandemic? Mental health trajectories, risk and protective factors in an Australian longitudinal study
Source: Aust N Z J Psychiatry. 2024 Jan 11;58(5):435–45. doi: 10.1177/00048674231223690 (PMC11055410; doi:10.1177/00048674231223690)
Supplement: sj-docx-1-anp-10.1177_00048674231223690 – Supplemental material for What set some young adults apart during the COVID-19 pandemic? Mental health trajectories, risk and protective factors in an Australian longitudinal study [file sj-docx-1-anp-10.1177_00048674231223690.docx]

# Supplementary Materials

Appendix S1. Missing data

**Table S1.** Number of respondents and non-respondents on the GAD-7 and PHQ-9 questionnaires at each follow-up survey wave

**Table S2.** Number of participants with one or more survey completions

**Table S3.** Sociodemographic differences between participants who completed baseline only and participants who completed one or more follow-up surveys

Appendix S2. Participant recruitment, eligibility and reimbursement procedures

Appendix S3. Unconditional growth model time score fit and GMM model indices

**Table S4.** Model fit statistics for GMM

Appendix S4. Descriptive results for depression and anxiety trends

**Figure S1.** Average GAD-7 scores across each survey timepoint

**Figure S2.** Average PHQ-9 scores across each survey timepoint

Appendix S5. Anxiety and depression trajectories with estimated means

**Figure S3.** Trajectories for symptoms of anxiety (GAD-7) including estimated means and N (%) for each class

**Figure S4.** Trajectories for symptoms of depression (PHQ-9) including estimated means and N (%) for each class

Appendix S6. Multinomial logistic regression analyses

**Table S5.** Univariate GAD-7 results

**Table S6.** Univariate PHQ-9 results

**Table S7.** Multivariate GAD-7 results

**Table S8.** Multivariate PHQ-9 results

**Appendix S1: Missing data**

While 758 participants were eligible for inclusion (aged 18-34 years) in the study at baseline, 105 participants (13.9%) were excluded due to incomplete or non-responses on baseline GAD-7 and PHQ-measures. The final analysed sample resulted in a total of 653 participants at baseline. Survey completions at all subsequent survey timepoints were voluntary and are detailed in Table S1. Approximately three quarters (73.8%) of the sample completed two or more surveys (Table S2). Missing data at follow-up timepoints were handled using full information maximum likelihood estimation in Mplus to provide unbiased estimates of data missing at random (Muthén and Muthén, 2017). Comparisons of sociodemographic characteristics between participants who completed the baseline only and participants who completed one or more follow-up surveys is provided in Table S3, using binomial logistic regression to assess significant differences. Participants who were older, had higher household income, living in Victoria, identified as female (compared to male) and were employed full-time or part-time at baseline (compared to unemployed) were less likely to *only* complete the baseline survey, relative to those who completed one or more follow-up surveys.

**Table S1.** Number of respondents and non-respondents on the GAD-7 and PHQ-9 questionnaires at each follow-up survey wave (total N = 653)

|  | GAD-7 completed | GAD-7 not completed | PHQ-9 completed | PHQ-9 not completed |
| --- | --- | --- | --- | --- |
| Wave 2 | 374 (57.3%) | 279 (42.7%) | 374 (57.3%) | 279 (42.7%) |
| Wave 3 | 399 (61.1%) | 254 (38.9%) | 399 (61.1%) | 254 (38.9%) |
| Wave 4 | 322 (49.3%) | 331 (50.7%) | 322 (49.3%) | 331 (50.7%) |

**Table S2.** Number of participants (%) with one or more survey completions (total N = 653)

|  | GAD-7 | PHQ-9 |
| --- | --- | --- |
| 1 completion (baseline) | 171 (26.2%) | 171 (26.2%) |
| 2 completions | 113 (17.3%) | 113 (17.3%) |
| 3 completions | 125 (19.1%) | 125 (19.1%) |
| 4 completions | 244 (37.4%) | 244 (37.4%) |

**Table S3.** Sociodemographic differences between participants who completed baseline only and participants who completed one or more follow-up surveys

|  | Baseline only  (N = 171)  N (%) or M (SD) | Baseline and 1+ follow-ups  (N = 482)  N (%) or M (SD) |  | |
| --- | --- | --- | --- | --- |
|  |  |  | OR (CI) | *p* |
| Participant gender |  |  |  |  |
| Male (reference) | 54 (31.6%) | 87 (18.0%) |  |  |
| Female | 111 (64.9%) | 382 (79.3%) | **0.47 (0.31-0.70)** | **<.001**** |
| Non-binary | 4 (2.3%) | 11 (2.3%) | 0.59 (0.16-1.81) | 0.380 |
| Other | 2 (1.2%) | 2 (0.4%) | 1.61 (0.19-13.7) | 0.638 |
| Age | 26.91 (4.71) | 28.08 (4.25) | **0.94 (0.91-0.98)** | **0.003**** |
| LGBTQIA+ | 41 (24.1%) | 120 (24.9%) | 0.96 (0.63-1.43) | 0.840 |
| State/Territory |  |  |  |  |
| NSW | 91 (53.2%) | 204 (42.3%) | 1.16 (0.74-1.81) | 0.525 |
| VIC | 41 (24.0%) | 177 (36.7%) | 0.60 (0.36-0.99) | **0.046**** |
| Other (reference) | 39 (22.8%) | 101 (21.0%) |  |  |
| English as a second language | 21 (12.5%) | 55 (11.8%) | 1.06 (0.61-1.80) | 0.818 |
| Employment |  |  |  |  |
| Employed full- or part-time | 94 (55.0%) | 307 (63.7%) | **0.46 (0.28-0.75)** | **0.002**** |
| Unemployed (reference) | 33 (19.3%) | 49 (10.2%) |  |  |
| University educated | 120 (70.2%) | 377 (78.2%) | 0.67 (0.41-1.12) | 0.121 |
| Lifetime mental health disorder | 68 (49.3%) | 255 (58.2%) | 0.70 (0.47-1.02) | 0.065 |
| Pre-pandemic stressful events | 1.03 (1.24) | 0.94 (1.25) | 1.06 (0.92-1.22) | 0.404 |
| Household income at Wave 1 |  |  | **0.83 (0.73-0.94)** | **0.003**** |
| <$300 | 14 (8.8%) | 10 (2.3%) |  |  |
| $300-$575 | 24 (15.1%) | 38 (8.8%) |  |  |
| $575-$1075 | 27 (17.0%) | 71 (16.4%) |  |  |
| $1075-$1700 | 32 (20.1%) | 105 (24.2%) |  |  |
| $1700-$2400 | 20 (12.6%) | 100 (23.0%) |  |  |
| >$2400 | 42 (26.4%) | 110 (25.3%) |  |  |

**Appendix S2: Participant recruitment, eligibility and reimbursement procedures**

Participants across all Australian states and territories were recruited via targeted social media advertisements (e.g., Facebook), community websites and physical flyers. To reach underserved populations, participants were also recruited via charitable organisations providing support and services to people experiencing, or at risk of, homelessness, housing issues and social disadvantage. In these instances, hard-copy flyers, consent information and surveys were administered on the service providers’ premises at each wave. To be eligible to partake in the *Alone Together Study*, participants were aged ≥18 years, currently residing in Australia and had sufficient English proficiency to understand the survey and consent procedures. Participants who completed the baseline survey were invited to participate in follow-up surveys via emails, phone calls, text messages and through original service providers. Surveys were hosted on Qualtrics, a secure online survey platform. Participants provided their informed and active written consent before commencing the 30–60-minute surveys. Following the completion of each survey wave, participants were entered into an optional prize draw to win one of two $250 vouchers.

**Appendix S3: Unconditional growth model time score fit and GMM model indices**

**Time score determination**: We tested different specifications of time scores (linear, quadradic and freely estimated) on unconditional growth models (i.e., single group, no class specifications) to determine the best fitting time structure for each outcome. In this case, linear models indicate that the slope reflects a change in the outcome over a 1-year interval, quadratic time scores indicate that the 1-year change in outcomes accelerates or decelerates at each interval (depending on the function of the quadratic term), and free time scores indicate that the slope reflects the overall change from baseline to 24-month follow up, allowing the data to account for any non-linear growth occurring at post-intervention and 12-month occasions (i.e., baseline time is fixed at 0, and 18-months at 1, but timepoints in-between are free). For both outcomes, freely estimated time scores provided the best model fit statistics by way of Akaike Information Criterion (AIC), Bayesian Information Criterion (BIC) and sample size-adjusted BIC (ssBIC). Model fit statistics for the best fitting unconditional growth models, and their type of time scores are reported in Table S4.

**Class selection justification:** Models with increasing numbers of classes (1-6 classes) were fit to the data and the optimal number of latent classes for each outcome variable were determined on the basis of parsimony, interpretability of classes, and model fit statistics, including the AIC, BIC and ssBIC, where lower values indicated a better model fit. The entropy statistic was used as a measure of classification accuracy, with values closer to 1 indicating stronger accuracy (Muthen, 2004). We ruled out models with trajectory classes containing less than 5% of the sample on statistical grounds. Statistically, estimates of parameters related to those classes with under 5% of the sample may become less stable and reliable (Nylund et al., 2007). This instability can result in imprecise parameter estimates, which can affect the validity of a model and the interpretation of the results. Based on model fit statistics (outlined in Table S4), and class interpretability, the 4- and 3-class models were chosen for anxiety and depression, respectively.

**Table S4.** Fit indices GMM models for GAD-7 and PHQ-9 (intercept freely estimated, slope constrained, free time)

| Measure | Number of  Classes | AIC | BIC | ssBIC | LL | Entropy Index | Smallest class in sample (%) |
| --- | --- | --- | --- | --- | --- | --- | --- |
| **GAD-7** |  |  |  |  |  |  |  |
|  | 2 | 10298.58 | 10338.914 | 10310.339 | -5140.29 | 0.561 | 44.10% |
|  | 3 | 10244.454 | 10307.196 | 10262.746 | -5108.227 | 0.656 | 4.75% |
|  | **4** | **10234.202** | **10305.907** | **10255.107** | **-5101.101** | **0.679** | **5.05%** |
|  | 5 | 10227.432 | 10317.063 | 10253.563 | -5093.716 | 0.699 | 4.44% |
|  | 6 | 10229.487 | 10323.6 | 10256.925 | -5098.743 | 0.703 | 4.75% |
| **PHQ-9** | | |  |  |  |  |  |
|  | 2 | 10600.1 | 10644.9 | 10613.2 | -5290.06 | 0.527 | 48.4% |
|  | **3** | **10555.67** | **10618.4** | **10574** | **-5263.83** | **0.610** | **11.18%** |
|  | 4 | 10535.9 | 10612.1 | 10558.1 | -5250.95 | 0.743 | 2.30% |
|  | 5 | 10527.1 | 10621.3 | 10554.6 | -5242.57 | 0.629 | 3.06% |
|  | 6 | 10526.7 | 10629.7 | 10556.7 | -5240.33 | 0.615 | 1.07% |

**Appendix S4: Descriptive results for anxiety and depressive symptom trends**

Our study used growth mixture models to examine changes in young adults’ mental health over two years of the COVID-19 pandemic. Across all survey time points, changes in average GAD-7 and PHQ-9 scores fell within the asymptomatic to mild symptom range. Graphical representations of raw means with SEM error bars are provided in Figure S1 and S2. Average mental health symptom scores must be considered within the context of competing COVID-19-related lockdowns, infection rates, border closures and infection-control measures. Average GAD-7 and PHQ-9 scores were initially elevated at Wave 1 between July to December 2020 following, and during, widespread lockdowns and restrictions across Australia. Scores decreased at Wave 2 between January to June 2021 during a period of reduced lockdowns and restrictions. Scores increased again at Wave 3 between July to December 2021 during strict widespread lockdowns and high infection rates across New South Wales and Victoria. Symptoms subsequently decreased at Wave 4 between January to June 2022, marked by easing restrictions, State border openings and reduced COVID-19 infection rates.

**Figure S1.** Average GAD-7 scores across each survey timepoint

**Figure S2.** Average PHQ-9 scores across each survey timepoint

**Appendix S5: Anxiety and depression trajectories with estimated means**

**Figure S3.** Trajectories for symptoms of anxiety (GAD-7) including estimated means and N (%) for each class


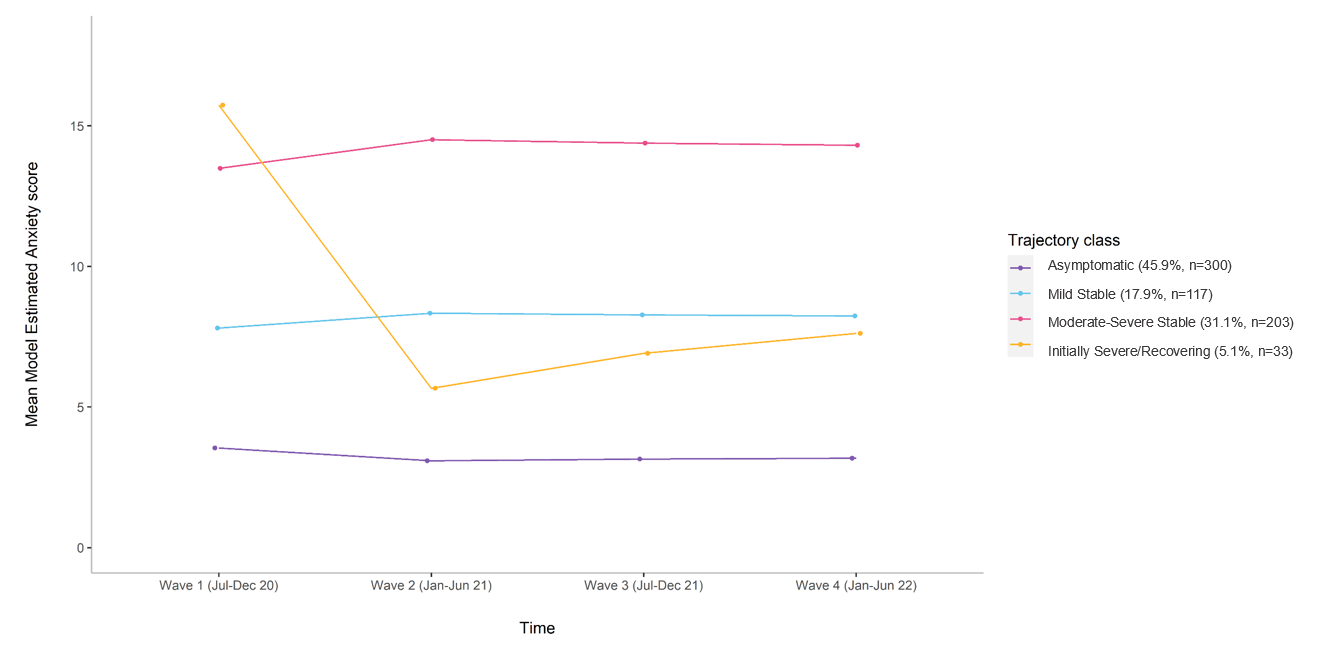


**Figure S4.** Trajectories for symptoms of depression (PHQ-9) including estimated means and N (%) for each class

**
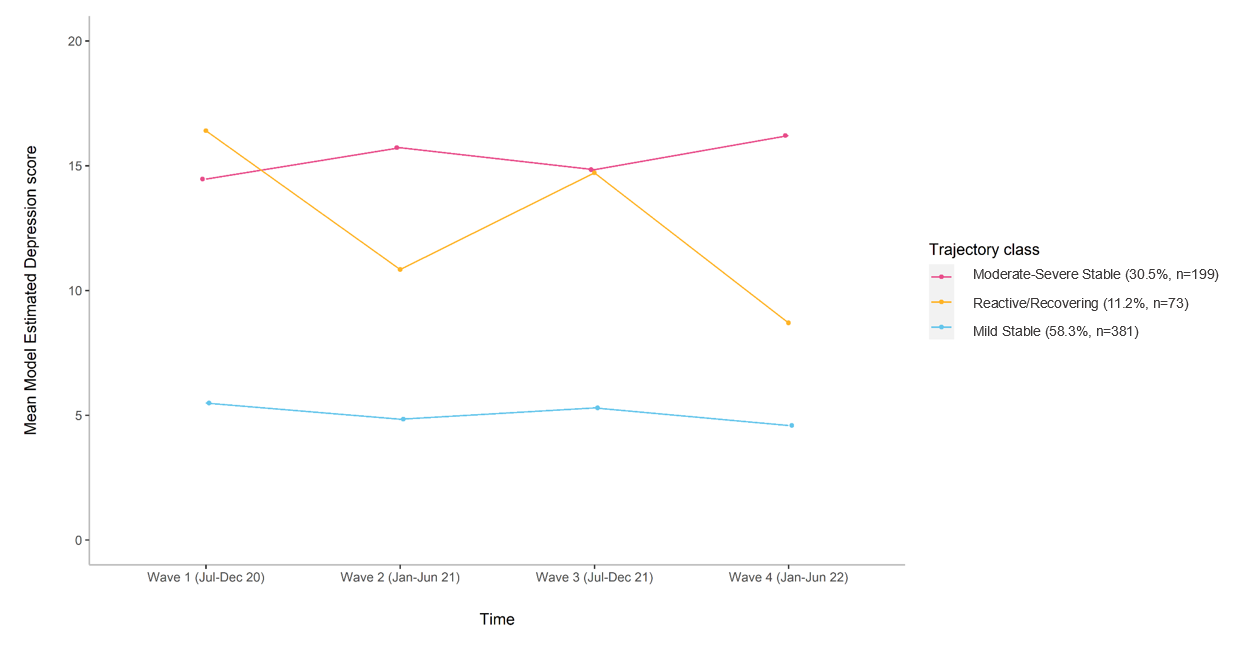
**

**Appendix S6: Multinomial Logistic Regression Analyses**

**Table S5.** Univariate GAD-7 – Asymptomatic reference group

| Variable (Reference group) | Mild Stable  (17.9%) | | Mode-Severe Stable  (31.1%) | | Initially Severe / Recovering (5.1%) | |
| --- | --- | --- | --- | --- | --- | --- |
|  | OR (95% CI) | p | OR (95% CI) | p | OR (95% CI) | p |
| Age | 0.99 (0.94 – 1.04) | 0.653 | 0.95 (0.91 – 0.99) | **0.020**** | 0.91 (0.84 – 0.99) | **0.023**** |
| Gender: Female (Male) | 2.11 (1.20 – 3.71) | **0.009**** | 1.90 (1.21 – 2.98) | **0.006**** | 1.34 (5.57 – 3.24) | 0.511 |
| Gender: Non-binary (Male) | 1.15  (1.22 – 1.09) | 0.901 | 5.03  (1.42 – 1.79) | **0.012*** | 5.93  (9.20 – 3.83) | 0.061 |
| LGBTQIA+ (Not LGBTQIA+) | 1.37 (0.82 – 2.28) | 0.225 | 1.91 (1.27 – 2.89) | **0.002**** | 1.81 (0.82 – 4.00) | 0.145 |
| Tertiary education (No tertiary education) | 0.67 (1.35 – 1.29) | 0.228 | 0.49 (0.29 – 0.84) | **0.010**** | 0.41 (0.15 – 1.10) | **0.077*** |
| Income | 1.07 (0.92 – 1.26) | 0.370 | 0.84 (0.74 – 0.95) | **0.007**** | 0.89 (0.69 – 1.15) | 0.360 |
| State: NSW (Other) | 0.88 (0.48 – 1.58) | 0.661 | 1.03 (0.65 – 1.63) | **0.891** | 0.52 (0.18 – 1.51) | **0.230** |
| State: Victoria (Other) | 1.86  (1.03 – 3.35) | **0.040**** | 1.05  (0.64 – 1.74) | **0.849** | 1.98  (0.78 – 5.02) | **0.150** |
| Lifetime mental health disorder (No) | 2.86 (1.78 – 4.61) | **<.001**** | 3.81 (2.53 – 5.73) | **<.001**** | 1.76 (0.83 – 3.73) | 0.138 |
| Pre-pandemic stressful events | 1.07 (0.88 – 1.30) | 0.511 | 1.38 (1.19 – 1.61) | **<.001**** | 1.32 (1.01 – 1.74) | **0.044**** |
| Increase in income (no change) | 1.12 (0.54 – 2.33) | 0.766 | 1.54 (0.82 – 2.89) | **0.176** | 0.38 (0.05 – 2.97) | 0.358 |
| Reduction in income (no change) | 0.63 (0.34 – 1.16) | 0.140 | 1.65 (1.06 – 2.56) | **0.026**** | 1.46 (0.63 – 3.37) | 0.379 |
| Employment disruptions (none) | 0.72 (0.44 – 1.18) | 0.194 | 1.06 (0.72 – 1.55) | 0.776 | 1.49 (0.71 – 3.13) | 0.289 |
| Housing disruptions (none) | 0.98 (0.56 – 1.71) | 0.946 | 1.06 (0.67 – 1.67) | 0.810 | 1.04 (0.43 – 2.52) | 0.936 |
| Social gains (no change) | 1.18 (0.52 – 2.68) | 0.697 | 1.00 (0.50 – 2.00) | 0.991 | 1.03 (0.24 – 4.38) | 0.968 |
| Social impairment (no change) | 1.37 (0.78 – 2.38) | 0.273 | 1.31 (0.83 – 2.06) | 0.241 | 1.41 (0.55 – 3.61) | 0.475 |
| Infection-control measures (none) | 1.52 (0.97 – 2.39) | **0.069*** | 0.93 (0.63 – 1.38) | 0.731 | 1.54 (0.74 – 3.20) | 0.251 |
| National stringency | 0.95 (0.89 – 1.01) | 0.115 | 0.95 (0.90 – 1.00) | **0.061*** | 0.91 (0.82 – 1.02) | 0.113 |

**Table S6.** Univariate PHQ-9 – Mild stable reference group

| Variable (Reference group) | Mode-Severe Stable  (30.5%) | | Reactive / Recovering (11.2%) | |
| --- | --- | --- | --- | --- |
|  | OR (95% CI) | p | OR (95% CI) | p |
| Age | 0.95 (0.92 – 0.99) | **0.016**** | 0.93 (0.88 – 0.98) | **0.012**** |
| Gender: Female (Male) | 1.25 (0.82 – 1.90) | 0.308 | 1.92 (0.94 – 3.90) | **0.073*** |
| Gender: Non-binary (Male) | 4.13  (1.14 – 14.93) | **0.030**** | 9.24  (2.00 – 42.75) | 0.004** |
| LGBTQIA+ (Not LGBTQIA+) | 2.38 (1.61 – 3.53) | **<.001**** | 2.39 (1.38 – 4.14) | **0.002**** |
| Tertiary education (No tertiary education) | 0.52 (1.32 – 0.85) | **0.010**** | 0.81 (0.37 – 1.76) | 0.600 |
| Income | 0.75 (0.66 – 0.85) | **<.001**** | 0.77 (0.64 – 0.93) | **0.006**** |
| State: NSW (Other) | 0.95 (0.60 – 1.49) | **0.820** | 0.60 (0.32 – 1.16) | **0.128** |
| State: Victoria (Other) | 1.14  (0.71 – 1.84) | **0.596** | 0.98  (0.51 – 1.88) | **0.957** |
| Lifetime mental health disorder (No) | 2.69 (1.83 – 3.96) | **<.001**** | 2.75 (1.56 – 4.84) | **<.001**** |
| Pre-pandemic stressful events | 1.37 (1.19 – 1.59) | **<.001**** | 1.40 (1.15 – 1.70) | **<.001**** |
| Increase in income (no change) | 1.05 (0.57 – 1.95) | **0.877** | 1.15 (1.45 – 2.93) | 0.765 |
| Reduction in income (no change) | 1.71 (1.11 – 2.63) | **0.015**** | 2.34 (1.29 – 4.25) | **0.005**** |
| Employment disruptions (none) | 1.06 (0.73 – 1.55) | 0.748 | 1.36 (0.80 – 2.31) | 0.250 |
| Housing disruptions (none) | 1.06 (0.68 – 1.66) | 0.792 | 1.42 (0.79 – 2.56) | 0.244 |
| Social gains (no change) | 1.98 (1.04 – 3.74) | **0.037**** | 1.85 (0.55 – 6.19) | 0.321 |
| Social impairment (no change) | 1.55 (0.99 – 2.42) | **0.054*** | 3.42 (1.51 – 7.76) | **0.003**** |
| Infection-control measures (none) | 1.16 (0.80 – 1.69) | 0.429 | 1.79 (1.07 – 2.98) | **0.026**** |
| National stringency | 0.95 (0.91 – 1.01) | **0.087*** | 0.94 (0.87 – 1.01) | 0.107 |

**Table S7.** Multivariate GAD-7 – Asymptomatic reference group

|  | Model 1 ^$^ | | | | | | Model 2 ^$$^ | | | | | | Model 3 ^$$$^ | | | | | |
| --- | --- | --- | --- | --- | --- | --- | --- | --- | --- | --- | --- | --- | --- | --- | --- | --- | --- | --- |
| Variable (Reference group) | Mild Stable  (17.9%) | | Mod-Severe Stable  (31.1%) | | Initially Severe / Recovering (5.1%) | | Mild Stable  (17.9%) | | Mod-Severe Stable  (31.1%) | | Initially Severe / Recovering (5.1%) | | Mild Stable  (17.9%) | | Mod-Severe Stable  (31.1%) | | Initially Severe / Recovering (5.1%) | |
|  | OR (95% CI) | p | OR (95% CI) | p | OR (95% CI) | p | OR (95% CI) | p | OR (95% CI) | p | OR (95% CI) | p | OR (95% CI) | p | OR (95% CI) | p | OR (95% CI) | p |
| Age | 0.99 (0.94 – 1.05) | 0.841 | 0.98 (0.93 – 1.03) | **0.435** | 0.91 (0.83 – 1.01) | **0.065*** | 0.99 (0.93 – 1.06) | 0.830 | 0.96 (0.91 – 1.02) | **0.158** | 0.89 (0.80 – 0.99) | **0.029**** | 1.01 (0.94 – 1.09) | 0.778 | 0.96 (0.90 – 1.02) | **0.185** | 0.89 (0.79 – 1.00) | **0.047**** |
| Gender: Female (Male) | 2.03 (1.11 – 3.71) | **0.021**** | 2.47 (1.48 – 4.12) | **<.001**** | 1.53 (0.58 – 4.01) | 0.388 | 1.48 (0.71 – 3.11) | 0.296 | 2.16 (1.09 – 4.28) | **0.026**** | 1.53 (0.46 – 5.15) | 0.490 | 1.24 (0.52 – 2.97) | 0.631 | 1.60 (0.72 – 3.57) | 0.249 | 1.10 (0.29 – 4.23) | 0.889 |
| Gender: Non-binary (Male) | 1.34  (0.13 – 14.16) | 0.807 | 3.22  (0.70 – 14.77) | 0.132 | 4.59  (0.56 – 37.33) | 0.154 | 1.27  (0.27 – 16.60) | 0.854 | 1.24  (0.18 – 8.64) | 0.828 | 4.68  (0.39 – 56.33) | 0.224 | 0.90  (0.06 – 14.62) | 0.943 | 0.49  (0.06 – 4.13) | 0.517 | 2.83  (0.21 – 37.78) | 0.432 |
| LGBTQIA+ (Not LGBTQIA+) | 1.18 (0.67 – 2.08) | 0.559 | 1.74 (1.09 – 2.77) | **0.019**** | 1.55 (0.64 – 3.74) | 0.328 | 0.91 (0.47 – 1.76) | 0.787 | 1.77 (1.04 – 3.01) | **0.034**** | 0.90 (0.31 – 2.61) | 0.842 | 0.89 (0.44 – 1.83) | 0.758 | 1.94 (1.09 – 3.44) | **0.024**** | 1.09 (0.37 – 3.22) | 0.880 |
| Tertiary education (No tertiary education) | 0.54 (0.25 – 1.16) | 0.117 | 0.53 (0.28 – 1.01) | **0.054*** | 0.58 (0.19 – 1.84) | 0.359 | 0.65 (0.28 – 1.52) | 0.324 | 0.94 (0.44 – 1.99) | 0.870 | 0.71 (0.19 – 2.60) | 0.605 | 0.71 (0.28 – 1.79) | 0.464 | 0.92 (0.41 – 2.05) | 0.834 | 0.71 (0.18 – 2.84) | 0.630 |
| Income | 1.10 (0.93 – 1.31) | 0.251 | 0.88 (0.77 – 1.02) | **0.085*** | 1.00 (0.76 – 1.32) | 0.993 | 1.10 (0.91 – 1.34) | 0.333 | 0.90 (0.77 – 1.06) | 0.216 | 1.01 (0.75 – 1.36) | 0.950 | 0.96 (0.77 – 1.20) | 0.741 | 0.96 (0.79 – 1.17) | 0.711 | 1.05 (0.75 – 1.46) | 0.786 |
| State – NSW (Other) | 0.79 (0.42 – 1.49) | 0.459 | 1.00 (0.60 – 1.69) | **0.987** | 0.50 (0.17 – 1.50) | **0.218** | 0.95 (0.46 – 1.98) | 0.896 | 1.30 (0.71 – 2.38) | **0.390** | 0.77 (0.23 – 2.56) | **0.667** | 0.72 (0.33 – 1.57) | 0.412 | 1.26 (0.65 – 2.47) | **0.492** | 0.56 (0.16 – 1.99) | **0.366** |
| State – Victoria (Other) | 1.52  (0.80 – 2.87) | 0.199 | 0.94  (0.54 – 1.65) | **0.827** | 1.61  (0.60 – 4.34) | **0.350** | 2.05  (1.00 – 4.21) | **0.051*** | 1.29  (0.68 – 2.45) | **0.429** | 2.28  (0.73 – 7.16) | **0.157** | 1.82  (0.84 – 3.94) | 0.127 | 1.39  (0.68 – 2.85) | **0.362** | 1.75  (0.52 – 5.92) | **0.36** |
| Lifetime mental health disorder (No) |  |  |  |  |  |  | 2.44 (1.45 – 4.09) | **<.001**** | 3.01 (1.89 – 4.18) | **<.001**** | 1.14 (0.48 – 2.68) | 0.765 | 2.62 (1.48 – 4.64) | **<.001**** | 2.97 (1.78 – 4.94) | **<.001**** | 1.37 (0.54 – 3.45) | 0.506 |
| Pre-pandemic stressful events |  |  |  |  |  |  | 1.04 (0.81 – 1.34) | 0.76 | 1.35 (1.10 – 1.66) | **0.004**** | 1.27 (0.90 – 1.79) | **0.180** | 0.98 (0.72 – 1.34) | 0.920 | 1.44 (1.13– 1.83) | **0.003**** | 1.27 (0.86 – 1.88) | **0.224** |
| Increase in income (no change) |  |  |  |  |  |  |  |  |  |  |  |  | 1.21 (0.47 – 3.16) | 0.690 | 1.41 (0.62 – 3.18) | **0.414** | 0.30 (0.03 – 2.87) | 0.293 |
| Reduction in income (no change) |  |  |  |  |  |  |  |  |  |  |  |  | 0.44 (0.18 – 1.06) | **0.068*** | 1.87 (0.96 – 3.63) | **0.065*** | 1.07 (0.34 – 3.35) | 0.913 |
| Employment disruptions (none) |  |  |  |  |  |  |  |  |  |  |  |  | 0.84 (0.44 – 1.61) | 0.600 | 0.72 (0.41 – 1.28) | 0.262 | 1.86 (0.71 – 4.83) | 0.205 |
| Housing disruptions (none) |  |  |  |  |  |  |  |  |  |  |  |  | 0.69 (0.33 – 1.45) | 0.327 | 0.75 (0.40 – 1.43) | 0.383 | 0.79 (0.25 – 2.50) | 0.688 |
| Social gains (no change) |  |  |  |  |  |  |  |  |  |  |  |  | 1.02 (0.34 – 3.08) | 0.968 | 0.83 (0.29 – 2.32) | 0.717 | 1.46 (0.27 – 7.85) | 0.659 |
| Social impairment (no change) |  |  |  |  |  |  |  |  |  |  |  |  | 1.01 (0.51 – 2.00) | 0.972 | 1.14 (0.61 – 2.13) | 0.688 | 0.83 (0.26 – 2.65) | 0.750 |
| Infection-control measures (none) |  |  |  |  |  |  |  |  |  |  |  |  | 2.18 (1.23 – 3.86) | **0.007**** | 0.57 (0.33 – 0.99) | **0.047**** | 1.90 (0.78 – 4.63) | 0.159 |
| National stringency |  |  |  |  |  |  |  |  |  |  |  |  | 0.96 (0.86 – 1.07) | 0.463 | 0.98 (0.89 – 1.08) | 0.707 | 0.81 (0.66 – 0.99) | **0.043**** |

^$^ Model 1: results for all sociodemographic variables
^$$^ Model 2: results for lifetime mental health disorder and pre-pandemic stressful events, adjusting for Model 1 variables
^$$$^ Model 3: results for all COVID-19-related stressors, adjusting for Model 1 and Model 2 variables

**Table S8.** Multivariate PHQ-9 – Mild stable reference group

|  | Model 1 ^$^ | | | | Model 2 ^$$^ | | | | Model 3 ^$$$^ | | | |
| --- | --- | --- | --- | --- | --- | --- | --- | --- | --- | --- | --- | --- |
| Variable (Reference group) | Mod-Severe Stable  (30.5%) | | Reactive / Recovering (11.2%) | | Mod-Severe Stable  (30.5%) | | Reactive / Recovering (11.2%) | | Mod-Severe Stable  (30.5%) | | Reactive / Recovering (11.2%) | |
|  | OR (95% CI) | p | OR (95% CI) | p | OR (95% CI) | p | OR (95% CI) | p | OR (95% CI) | p | OR (95% CI) | p |
| Age | 0.98 (0.93 – 1.02) | **0.312** | 0.95 (0.89 – 1.02) | 0.155 | 0.96 (0.91 – 1.02) | **0.158** | 0.92 (0.85 – 0.99) | **0.033**** | 0.95 (0.89 – 1.01) | **0.073*** | 0.94 (0.86 – 1.02) | **0.128** |
| Gender: Female (Male) | 1.44 (0.90 – 2.30) | 0.133 | 2.61 (1.15 – 5.92) | **0.021**** | 1.21 (0.66 – 2.23) | 0.535 | 2.10 (0.76 – 5.84) | 0.153 | 0.81 (0.39 – 1.66) | 0.561 | 1.26 (0.41 – 3.88) | 0.691 |
| Gender: Non-binary (Male) | 1.91  (0.40 – 9.20) | 0.420 | 7.22  (1.23 – 42.27) | **0.028**** | 1.95  (0.19 – 20.23) | 0.578 | 9.14  (0.75 – 110.98) | 0.082 | 0.78  (0.07 – 9.20) | 0.847 | 4.35  (0.33 – 57.08) | 0.263 |
| LGBTQIA+ (Not LGBTQIA+) | 2.14 (1.38 – 3.32) | **<.001**** | 2.19 (1.18 – 4.09) | **0.013**** | 2.41 (1.46 – 3.98) | **<.001**** | 1.85 (0.92 – 3.73) | **0.086*** | 2.69 (1.56 –4.62) | **<.001**** | 1.91 (0.90 – 4.03) | **0.090*** |
| Tertiary education (No tertiary education) | 0.71 (0.39 – 1.28) | 0.254 | 1.13 (0.45 – 2.83) | 0.789 | 1.18 (0.59 – 2.37) | 0.635 | 1.42 (0.53 – 3.81) | 0.487 | 1.27 (0.61 – 2.69) | 0.529 | 1.18 (0.41 – 3.37) | 0.764 |
| Income | 0.80 (0.69 – 0.91) | **<.001**** | 0.81 (0.67 – 0.99) | **0.042**** | 0.80 (0.69 – 0.94) | **0.005**** | 0.85 (0.68 – 1.06) | 0.143 | 0.83 (0.69 – 1.00) | **0.051*** | 0.92 (0.71 – 1.19) | 0.530 |
| State: NSW (Other) | 1.06 (0.63 – 1.76) | **0.835** | 0.61 (0.30 – 1.25) | **0.174** | 1.37 (0.75 – 2.49) | **0.302** | 0.72 (0.33 – 1.58) | **0.408** | 1.18 (0.61 – 2.28) | 0.614 | 0.57 (0.24 – 1.32) | **0.189** |
| State: Victoria (Other) | 1.20  (0.70 – 2.04) | **0.514** | 0.90  (0.43 – 1.85) | **0.769** | 1.56  (0.84 – 2.88) | **0.157** | 1.03  (0.47 – 2.28) | **0.936** | 1.50  (0.76 – 2.97) | 0.247 | 0.78  (0.33 – 1.82) | **0.560** |
| Lifetime mental health disorder (No) |  |  |  |  | 2.10 (1.35 – 3.27) | **0.001**** | 1.92 (1.01 – 3.65) | **0.045**** | 2.26 (1.39 – 3.68) | **0.001**** | 1.82 (0.92 – 3.58) | **0.083*** |
| Pre-pandemic stressful events |  |  |  |  | 1.30 (1.07 – 1.58) | **0.008**** | 1.27 (0.99 – 1.65) | **0.064*** | 1.39 (1.10 – 1.76) | **0.005**** | 1.19 (0.89– 1.59) | **0.252** |
| Increase in income (no change) |  |  |  |  |  |  |  |  | 0.81 (0.36 – 1.80) | **0.603** | 0.69 (0.22 – 2.19) | 0.531 |
| Reduction in income (no change) |  |  |  |  |  |  |  |  | 1.43 (0.75 – 2.71) | **0.273** | 1.83 (0.81 – 4.13) | 0.146 |
| Employment disruptions (none) |  |  |  |  |  |  |  |  | 0.80 (0.46 – 1.38) | 0.428 | 1.29 (0.63 – 2.62) | 0.483 |
| Housing disruptions (none) |  |  |  |  |  |  |  |  | 0.57 (0.30 – 1.08) | **0.085*** | 1.32 (0.61 – 2.85) | 0.488 |
| Social gains (no change) |  |  |  |  |  |  |  |  | 2.51 (1.00 – 6.30) | **0.049**** | 2.87 (0.64 – 12.91) | 0.169 |
| Social impairments (no change) |  |  |  |  |  |  |  |  | 1.33 (0.73 – 2.40) | 0.353 | 2.71 (0.96 – 7.62) | **0.059*** |
| Infection-control measures (none) |  |  |  |  |  |  |  |  | 0.85 (0.52 – 1.39) | 0.511 | 1.56 (0.82 – 2.98) | 0.176 |
| National stringency |  |  |  |  |  |  |  |  | 0.95 (0.86 – 1.04) | 0.260 | 0.86 (0.74 – 0.99) | **0.038**** |

^$^ Model 1: results for all sociodemographic variables
^$$^ Model 2: results for lifetime mental health disorder and pre-pandemic stressful events, adjusting for Model 1 variables
^$$$^ Model 3: results for all COVID-19-related stressors, adjusting for Model 1 and Model 2 variables

**References**

Muthen B (2004) Latent variable analysis: Growth mixture modeling and related techniques for longitudinal data. In Handbook of quantitative methodology for the social sciences, ed. D. Kaplan, 345-68. Thousand Oaks, CA: Sage.

Muthén B and Muthén L (2017) Mplus. *Handbook of item response theory*. Chapman and Hall/CRC, pp.507-518.

Nylund KL, Asparouhov T and Muthén BO (2007) Deciding on the Number of Classes in Latent Class Analysis and Growth Mixture Modeling: A Monte Carlo Simulation Study. *Structural Equation Modeling: A Multidisciplinary Journal* 14(4): 535-569.
